# Multiple Scattered and Small Lesions of Lower Limbs: Idiopathic Calcinosis Cutis: A Case Report

**DOI:** 10.5704/MOJ.1803.008

**Published:** 2018-03

**Authors:** NA Ferdaus-Kamudin, NH Mohamed-Haflah

**Affiliations:** Department of Orthopaedics, Universiti Sultan Zainal Abidin, Kuala Terengganu, Malaysia; ^*^Department of Orthopaedics and Traumatology, Universiti Kebangsaan Malaysia, Cheras, Malaysia

**Keywords:** calcinosis cutis, idiopathic, multiple lesions of lower limbs, calcium deposits

## Abstract

Calcinosis cutis is a rare presentation and not many cases have been reported especially of idiopathic type. We are reporting a case of idiopathic calcinosis cutis of lower limbs in a 33-year old female who presented to our clinic for multiple painless swellings over her lower limbs for the past six months, without any history of trauma or infection. We have decided to observe her condition on regular follow-up and conservative management.

## Introduction

Calcinosis cutis is the uncommon and abnormal deposition of calcium in the skin at various parts of the body^1^. It is a rare presentation and very few cases have been reported especially for idiopathic calcinosis cutis^1^. The first case was reported by Virchow in 1855. Calcinosis cutis is divided into four main types according to aetiology: dystrophic, metastatic, iatrogenic and idiopathic^1^. We report a case of idiopathic calcinosis cutis of lower limbs.

## Case Report

A 33-year old female presented to our clinic for multiple painless swelling over her lower limbs for the past six months. She denied any history of trauma, insect bites or any suggestion of ongoing infection over her lower limbs. She had no joint pain and no constitutional symptoms. Her daily activity was normal.

**Fig. 1:**
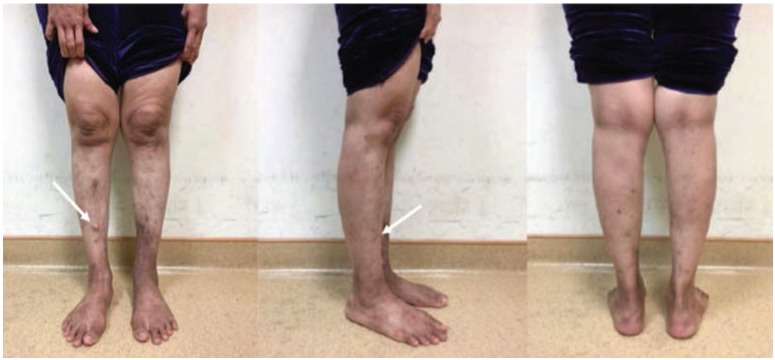
Multiple lesions over both lower limbs, scattered and small in sizes (largest lesion located over the right leg indicated by arrow).

**Fig. 2:**
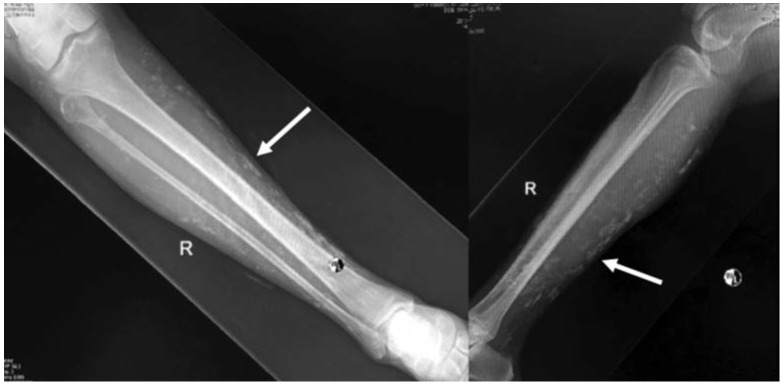
The radiographs of right tibia and fibula shows multiple calcification lesions within subcutaneous tissue, scattered and small in sizes.

**Fig. 3:**
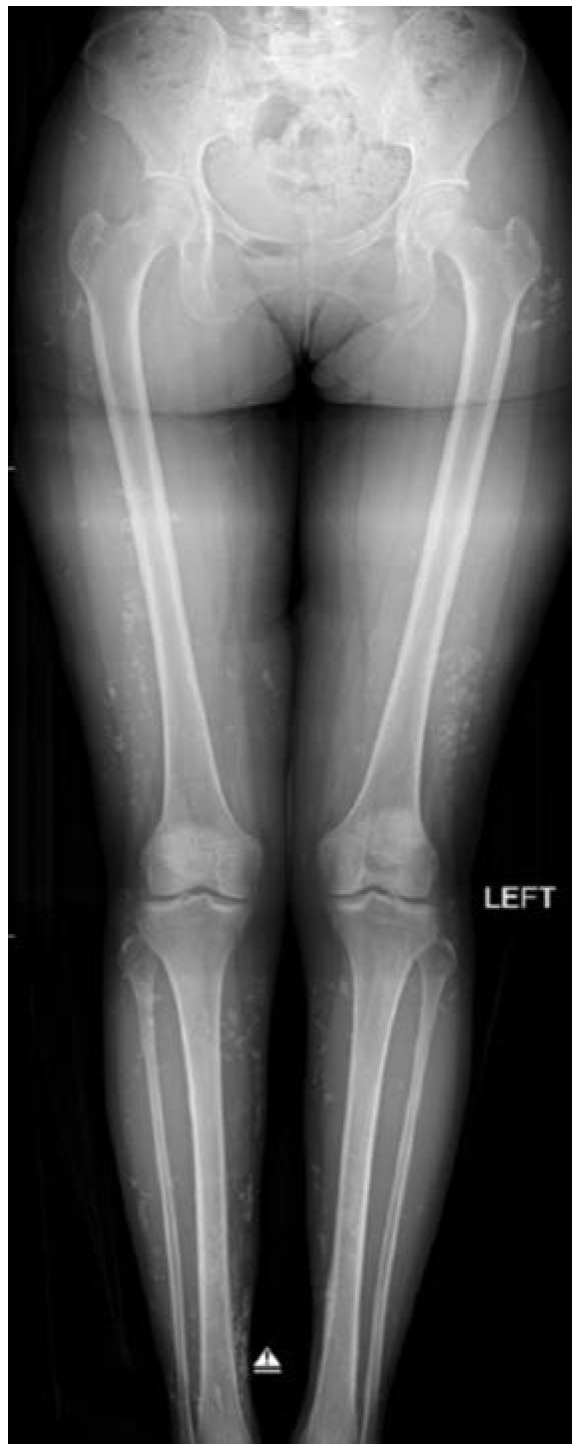
The scanogram of the lower limbs showing multiple calcification lesions within subcutaneous tissue.

The lesions over her lower limbs were multiple and of varying sizes (Fig. 1). The biggest lesion was over the distal third of the right leg of size 1cm x 1cm and hard in consistency. They were not attached to the skin or underlying structures, non-tender and no skin changes or prominence of superficial vessels. There were no similar lesion noted elsewhere on her body. Her general clinical examination was unremarkable. All joints were supple. Plain radiograph of lower limbs showed multiple calcification lesions within subcutaneous tissue (Fig. 2 and 3).

The laboratory parameters such as full blood count and renal profile were normal, as well as erythrocyte sedimentation rate (ESR) and C-reactive Protein marker (CRP). The urine protein index was 0.01 (normal range). Serum calcium, phosphate, magnesium and parathyroid hormone levels were normal.

Based on the radiological findings and unremarkable laboratory findings, we made the diagnosis of idiopathic calcinosis cutis of lower limbs. The patient was given subsequent follow-up to detect any new lesion or increase in size of the nodules over the limbs.

## Discussion

Calcinosis cutis is a disorder where calcium is deposited in the subcutaneous tissue, initially described by Virchow in 1855. It is divided into four types. The first type is dystrophic calcification, described as tissue destruction which is localized or widespread. The second is metastatic calcification, which shows aberrant calcium and phosphorus metabolism. If there is no sign of tissue damage or abnormal metabolic activity, it is known as idiopathic calcification, which is the third type. The last type is iatrogenic calcinosis secondary to treatment or procedures^1^.

Very few reports on idiopathic calcinosis cutis are available. Jatana *et al* described examples of idiopathic calcinosis such as calcinosis at scrotum and penis, milia-like idiopathic calcinosis cutis, subepidermal calcified nodule, tumoral calcinosis, calcinosis cutis circumscripta and calcinosis universalis^1^. Lee *et al* also described a classification of calcinosis cutis based on morphological pattern, divided into two main groups: with or without vascular involvement. Lesions without vascular involvement are divided into small scattered, nodular, tumoral and widespread. Calciphylaxis was the only example with vascular involvement^2^. In our case, the lesions were small scattered depositions of calcification within subcutaneous tissue.

Our case report is about a patient with idiopathic calcinosis cutis, which is one of the rare type of conditions^1^. Its pathogenesis is unclear. According to Lanka *et al*, abnormal metabolism of gamma-carboxy glutamic acid (GCGA) leads to its deposition in the skin causing the idiopathic type of calcinosis cutis. This unique amino acid is found normally in bones and deep tissue^3^. Metabolic and physical factors are also postulated in the development of calcinosis in the tissue. Metabolic involvement, hypercalcemia and/or hyperphosphatemia may lead to calcium-phosphate nucleation and crystalline precipitation. Physical factors such as following tissue damage may cause an influx of calcium ions leading to an elevation of intracellular calcium level. Tissue damage also will cause denatured proteins that will bind phosphate, which eventually lead to precipitation of calcium phosphate and crystalline formation^4^.

Our case falls into the category of idiopathic calcinosis cutis of lower limbs. The location of calcium deposition was found in subcutaneous tissue without any ulcer over the skin. We also had ruled out any co-existing conditions such as parathyroid disease, kidney failure or possible metastatic calcification, based on the history, physical examination, radiological and laboratory investigations. The underlying causes must be determined first before commencement of treatment. Histological examination reveals calcium deposition in the dermis, which sometimes might be surrounded by foreign-body giant cell reaction. In an area with tissue necrosis, calcium deposition may be detected in the vessels as well^4^.

Calcinosis cutis can be treated using medical therapy or surgical intervention. The medical therapy such as corticosteroid, probenecid, and colchicine have been used in some patients. The usage of magnesium or aluminium antacids as phosphate binders could be used in patients with hyperphosphatemia. To prevent bone turnover and prevent formation ectopic hydroxyapatite crystals, some reports suggest the usage of sodium etidronate and diphosphates^1^. Surgical excision is only indicated if the lesions are painful, infected or affecting limb function. However, it may lead to stimulation of more calcification which could make the condition worse with risk of recurrence^5^.

Since our patient had presented with painless, small scattered lesions, with no clinical sign of tissue destruction or ulceration, and normal functional limbs, we decided to observe progress of the lesions with regular follow-up.

We present this case to create awareness in orthopaedic colleagues regarding calcinosis cutis of lower limbs.

## References

[1] Jatana SK, Negi V, Das S (2012). A case of idiopathic calcinosis cutis. Med J Armed Forces India.

[2] Lee JH, Park HJ, Lee JY, Cho BK (2008). Case of dystrophic calcinosis cutis in epidermal cyst arising from verrucous epidermal nevus. J Dermatol..

[3] Lanka P, Lanka LR, Ethirajan N, Krishnaswamy B, Manohar U (2009). Idiopathic calcinosis cutis. Indian J Dermatol..

[4] Nunley JR Calcinosis cutis.

[5] Hawkins R, Mehta R (2016). A novel approach in the treatment of calcinosis cutis. J Clin Rheumatol..

